# Educational

**Published:** 1897-10

**Authors:** 


### EDITORIAL.

### Educational.

The time of beginning the work of the annual sessions of the dental colleges of the country is near at hand. From the statements of some of those intimately connected with the colleges, the indications are quite apparent that a larger number of students will enter for the coming year's work than ever before.

Two or three new colleges have been added within the past year. As the number of colleges increases and the classes become larger, more interest and enthusiasm attaches to the work. Faculties and boards of trustees are putting forth more effort than ever before to increase their facilities and improve their methods of work, all of which augurs well for the future of the profession. There never was so much nor so good textual and journalistic literature available for teachers and students, as at present.

There is a determined effort on the part of the majority, if not all of these institutions, to elevate the standard of dental education. This is manifest by the demand for higher preliminary or entrance requirements, as well as extending the curriculum of the dental course, and to do more thorough work upon it. There is also manifest a disposition to greater caution in the selection and qualifications of those who are to assume the responsibility of teachers.

It is with much gratification that we feel warranted in making reference to these evidences of progress, but while doing so we are not unmindful of the fact that the way is well open for the accomplishment of much that ought to be done if proper appreciation was entertained of the needs and progress of the times. But satisfaction may be entertained in this matter when we review what has been done in the past.

It is to be hoped that there will this year, be a much higher standard of literary requirements than ever before, and that more caution will be exercised in the admission of students, than hitherto.

